# Impact of Equine and Camel Piroplasmosis in Egypt: How Much Do We Know about the Current Situation?

**DOI:** 10.3390/pathogens12111318

**Published:** 2023-11-05

**Authors:** Bassma S. M. Elsawy, Mona S. Mahmoud, Carlos E. Suarez, Heba F. Alzan

**Affiliations:** 1 Parasitology and Animal Diseases Department, Veterinary Research Institute, National Research Center Dokki, Giza 12622, Egypt; bs.el-sawy@nrc.sci.eg (B.S.M.E.); monasaid3000@yahoo.com (M.S.M.); 2 Tick and Tick-Borne Diseases Research Unit, Veterinary Research Institute, National Research Center Dokki, Giza 12622, Egypt; 3 Department of Veterinary Microbiology and Pathology, College of Veterinary Medicine, Washington State University, Pullman, WA 99164, USA; 4 Animal Disease Research Unit, United States Department of Agricultural—Agricultural Research Service, Pullman, WA 99164, USA; carlos.suarez@usda.gov

**Keywords:** equine, camel, *piroplasma*, *Babesia*, tick, tick borne diseases, *Theileria*, diagnosis, microscopical examination, serological examination, PCR, treatment and control

## Abstract

Piroplasmosis is a global tick-borne disease caused by hemoprotozoan parasites, which causes high morbidity and substantial economic losses in farm animals. Equine and camel piroplasmosis causes significant losses worldwide and in Egypt. The multifactorial effects and overall impact of equine and camel piroplasmosis in Egypt remain poorly characterized. However, several *Babesia* and *Theileria* spp. as well as potential tick vectors affecting these two species have been identified in the country. Equine and camel piroplasmosis has been reported by all governates in the country. Thus, in this work, we intend to provide a broad depiction of the current approaches used for diagnosis and control and the impact of piroplasmosis on the equine and camel industries in Egypt. We also identified current gaps in knowledge that might help develop future research efforts towards improved intervention and control of equine and camel piroplasmosis. It is important to develop specific diagnostic tools suitable for the early and chronic diagnosis of this disease. Altogether, the current situation warrants the development of large-scale epidemiological studies in order to obtain an accurate estimate for equine and camel piroplasmosis to secure the highly needed food resources in the country.

## 1. Introduction

Ticks and tick-borne diseases such as babesiosis, theileriosis, and anaplasmosis pose a significant threat to animal and human health and can cause significant economic losses to the livestock industry, mainly in tropical and semitropical countries where they occur. These losses are partly attributed to the lack of sensitive, robust, cost-effective, and efficient diagnostic and preventive approaches that can detect and control the spread of infectious pathogens at the early stages of illness [1].

Tick-borne pathogens circulate throughout enzootic cycles, alternating between tick vectors and vertebrate hosts. Tick-borne diseases in livestock are the cause of multiple negative effects among animal holders, including the costs incurred for the implementation of preventive measures aimed at controlling these infections, the stresses caused by the loss of their animals, and the need for usually cumbersome administrative approvals. Therefore, improving the global control of animal tick-borne diseases and their vectors would contribute to better social welfare as well as improved meat production [2]. At least 60 tick-borne agents have been recognized as pathogenic to livestock; however, only a few of them are known, so far, to cause economic losses [3]. Moreover, it has been known for some time that ticks can be co-infected with more than one pathogen and transmit multiple pathogens simultaneously while taking blood meals from their hosts [1,4].

Piroplasmosis is an important tick-borne disease that can affect many vertebrate hosts from humans to bats, as well as farm animals such as ruminants, equines, and dromedaries. This disease is caused by blood-borne piroplasmids, mainly *Babesia* and *Theileria*. It occurs frequently in rural areas of developing countries, including Egypt, where there is still a huge reliance on working equines, which include horses, donkeys, mules, and ponies [5,6,7]. Particularly, camels are an economically significant multipurpose animal that has been utilized traditionally as an important source of milk, meat, and wool, and they are widely distributed in Africa, the Middle East, and Northern India [8,9]. In addition, there has recently been a steady increase in the number of camels slaughtered for meat consumption [10].

Piroplasmosis is biologically transmitted by several ticks of the genera *Dermacentor*, *Hyalomma*, and *Rhipicephalus* [11]. However, it can also be transmitted by direct inoculation of parasite-contaminated blood or blood products, or through the use of blood-contaminated fomites, including equipment such as needles, syringes, surgical instruments, dental equipment, and tattooing equipment, among others [12].

Equine piroplasmosis [EP] is mainly caused by *T. equi*, *B. caballi,* and the newly identified species, *T. haneyi* [7,12,13]. In camels, this disease may be caused mainly by *T. equi* and *B. caballi* [14,15,16,17]. Also, *B. bovis* and *B. bigemina* were reported [10,16,17,18], in addition to *T. annulata* and *T. ovis* [19], *Babesia vulpes*, *Babesia* sp., and *Theileria* sp. [16,17,20]. Also, *T. camelensis* is considered one of the causes of EP, but this is still unclear because of the gap in the studies regarding experimental infections and molecular characterization of this parasite [17]. The control and treatment of piroplasmosis mainly relies on the accurate diagnosis and identification of the causative agent by serological and molecular investigative techniques [21], and the availability of effective preventive and curative methods.

The aim of this study is to present available information about the different causative agents of equine and camel piroplasmosis circulating in Egypt by identifying current knowledge and research gaps, the diagnostic methods currently in use, the economic impact of equine and camel piroplasmosis, the applied control strategies, and the effects of the disease on the equine and camel populations in the country, along with a general review of the equine and camel industry in Egypt.

## 2. Data Collection and Analysis

### 2.1. Searching Strategy

The PubMed, Scopus, and ScienceDirect databases were searched for studies on piroplasm infecting equines and camels in Egypt published in English until July 2023. Various keywords were used for the search, including ticks, tick-borne diseases, *Babesia*, babesiosis, *Theileria*, and theileriosis. The keywords were used in combination with the animal species (camels, equines, horses, and donkeys) and detection methods (microscopical examination, serological examination, and molecular examination (PCR)) as well as “Egypt” (Table 1). To combine the entry terms, the Boolean operators “OR” and “AND” were used. In addition, the Egyptian Knowledge Bank’s website (http://www.ekb.eg, accessed in 2 July 2023) was searched to collect papers published in local Egyptian scientific journals. The Google Scholar search engine was used to ensure that the entire contents from all relevant publications, and not just abstracts, were included in the data gathering. The same keywords were used in all databases.

### 2.2. Data Extraction

Data from available studies on *Piroplasma* spp. infections in equines and camels in Egypt were separated and organized using a Microsoft Excel^®^ spreadsheet. The following information was extracted whenever available: study governorates, sample size, prevalence, diagnostic method, and detected *Piroplasma* spp.

## 3. An Overview of the Equine and Camel Industry in Egypt

The estimated current sizes of the target animal populations in Egypt include 120,000 camels and 85,000 horses [4,22].

### 3.1. Equines

About 230 farms in Egypt specialize in raising Arabian horses, and the Egyptian Agricultural Authority offers pedigree certifications for all horses sold to foreign nations that go back up to six generations, in addition to permanently marking all animals they possess (Freeze Marking). Also, a single office creates the formal documents required for export activities.

In Egypt, a number of horse breeders from various nations are invited to a competition that is held every year in the month of November. This event has a number of equestrian competitions and shows that are judged by an international committee. The organization of international festivals and contests that take place in Egypt has an important economic impact, since numerous visitors from foreign countries, including neighboring Arabic countries, are usually interested in attending these events, which also refreshes the tourist industry and drives horse trading [23].

Additionally, in many rural areas of Egypt, horses, donkeys, mules, and ponies are often used as working equids. These animals assist personnel in a variety of sectors, including agriculture and construction, help farmers in soil drilling and public transportation, and contribute to sustaining the livelihoods of millions of people [5,6].

### 3.2. Camels

Three species of camels can be found in Egypt: the one-humped Arabian camel [also known as dromedaries] (*Camelus dromedarius*), the Bactrian camel (*Camelus bactrianus*), which is a two-humped camel, and its wild counterpart (*Camelus ferus*) [19,24,25,26]. The one-humped camel *Camelus dromedarius*, or dromedary, is a domestic animal belonging to the Camelidae family and is widely distributed in the arid and semi-arid regions of Africa, Arabia, and western Asia, extending up to India [8]. The world’s current camel population is about 28 million heads, and 80% of them live in Africa, with 60% in the Horn of Africa. Arabian camels (Dromedaries) constitute 94% of the world’s camel population [22,27]. In Egypt, there are four distinct camel breeds, belonging to Camelus dromedarius, which differ phenotypically: the Sudani (often used for riding and racing), the Falahi or Baladi (used for transportation and agricultural work), the Maghrabi (used for both meat and milk), and the Mowallad (a hybrid of the two) [28].

Arabian camels significantly contribute to Egypt’s local economy and culture. They do so by producing milk and meat for human consumption, as well as wool. Regarding camel milk production, unfortunately in Egypt, camel milk is underestimated, and it does not seem to contribute significantly to the economy of the country [28], despite its high nutritional value. The camel contribution to meat production started to increase not only in Egypt but also in other developing countries [10], given the fact that camels are likely to have disease-resistance traits [28]. Additionally, camels serve as a mode of transportation, particularly in the desert which is widely distributed in Egypt; therefore, they are an important component of nomadic life. Camel rearing is primarily practiced for recreational and entertainment purposes in tourist areas such as the Luxor and Red Sea governorates [20]. In addition, camel racing is considered a popular traditional sport in many Arab countries, most notably in the Gulf region, and in Egypt, Bedouins of the South Sinai desert have kept up this tradition. To the Bedouins, the race is a way of keeping a traditional heritage alive. This race is considered an ancestral heritage and they are trying to preserve and renew it to hand it over from one generation to the next, which has been ongoing for at least the last 100 years [29].

Smallholders occasionally raise camels in the countryside, together with other animals, or on their own farms. They can also do so in desert pastures like those in the Sinai Peninsula, the northwest coastal region, and the Red Sea coast [18]. Between 2012 and 2015, Sudan and Ethiopia were the major sources of camels for Egypt, with more than 750,000 camel imports during this time [19,30]. In fact, the Food and Agriculture Organization [FAO] recorded an increase in the camel population in Egypt from 111,000 in 2010 to about 149,500 in 2017 [28]. Notably, Egypt needs to import large numbers of live camels because the high rate of slaughtering is resulting in the fast depletion of the stock of available animals [28].

## 4. Impact of Equine and Camel Piroplasmosis in Egypt

Since equines and camels are currently important resources for recreation and food production in Egypt, maintaining healthy populations of these species is critical. This diminishes the chances for the expansion of emerging zoonotic agents, such as *Babesia microti* and *B. divergens*, which may impact human health and create improved economic environments for the producers. In addition, uncontrolled camel piroplasmosis is also a threat to the production of critical food resources that can sustain the current high population growth rates in Egypt.

### 4.1. Equines

In rural areas of Egypt, the health and welfare of domestic equines are often neglected despite the high risk of contracting many infectious diseases, including African horse sickness, epizootic lymphangitis (EZL), rabies, trypanosomiasis, and piroplasmosis. Knowledge about the identification, management, and prevention of different infectious diseases is lacking in general [31].

Equine piroplasmosis, recognized as one of the most frequent infectious tick-borne diseases (TBDs) in equids, is caused by the hemoprotozoan parasites *T. equi, B. caballi,* and the newly identified species *T. haneyi* [12,13]. It is possible, however, that additional and likely lowly virulent equine *Babesia* and *Theileria* species will be identified in the future. Infections with *T. equi* and *B. caballi* cause severe economic losses in the equine industry due to the cost of treatment, especially in acutely infected horses. Additionally, the absence of appropriate treatments can lead to the death of the animals [6], and the infected and carrier equines are a common source of infection for ticks and other animals [16].

Importantly, EP manifests as acute and persistent infections. Clinical signs are not specific to EP and vary from lacking to severe, whereas signs in acute cases are characterized by fever, anemia, hemoglobinuria, jaundice, edema, and even death [32]. Furthermore, and because EP is also characterized by persistent infections, horses and donkeys may act as carriers for many years, particularly after *T. equi* infection [33]. It was found that *T. haneyi* causes milder clinical disease (variable fever, anemia) than *T. equi* in experimentally infected horses and is capable of superinfection with *T. equi* [34]. After the acute phase of the disease, asymptomatic horses may continue to be infected and these asymptomatic horses may become reservoirs of infectious organisms for the appropriate vectors of ticks [35]. Unfortunately, *T. haneyi* does not appear to be susceptible to imidocarb diproprionate (ID), although most equine infections with U.S. strains of *T. equi* can be treated with ID, and co-infections of horses with *T. equi* and *T. haneyi* reduce the effectiveness of ID against *T. equi*. So, the global importance of *T. haneyi* to equine health was recently shown through its resistance to ID and its interference with *T. equi* clearance by ID in some co-infected horses [34].

### 4.2. Camels

Although camels can tolerate harsh conditions, they can also be affected by climatic changes and by infections with different infectious diseases, including those caused by vector-borne hemopathogens, which frequently compromise the health and production of camels [20].

Camel piroplasmosis (CP) is an acute to chronic infectious disease with a worldwide distribution that causes high morbidity and substantial economic losses [18]. Similar to EP, CP can be caused by several *Theileria* and *Babesia* parasites, including *T. equi*, *B. caballi*, *B. bovis*, *B. bigemina*, among others [17]. Clinical symptoms include anemia, hemoglobinuria, muscle trembling, and decreases in body temperature to a subnormal level a few hours of before death in untreated cases [36].

Camel babesiosis, caused by several tick-borne *Babesia* sp., is marked by severe morbidity and substantial economic loss [15]. There is a lack of information about camel infections caused by *Babesia* species, which are of zoonotic importance in Egypt. One of the most significant *Babesia* species that affects humans is *Babesia microti*, which may spread through blood transfusion or organ transplantation [37]. Using molecular diagnostic methods and phylogenetic analysis of the discovered parasite, some researchers found *B. microti* infections in camel breeds in Halayeb and Shalateen in Upper Egypt [9]. This was a significant finding because the possible existence of camel reservoirs may represent a potential zoonotic risk to other animals and humans. In contrast to other animals, there is little knowledge of camels’ involvement in sustaining zoonotic tick-borne pathogens (TBPs), despite the importance of camels to human life in the country [9].

## 5. Competent Tick Vectors for Equine and Camel *Piroplasma* spp. Identified in Egypt

### 5.1. Equines

More than 30 different species of ticks are known to be vectors for *T. equi* or *B. caballi,* and these include the genera *Hyalomma, Rhipicephalus, Dermacentor, Amblyomma,* and *Haemaphysalis* spp. [11]. In Egypt, three species of ticks have been detected infesting equids, i.e., *Hyalomma dromedarii, Hyalomma excavatum*, and *Rhipicephalus annulatus* [38]. However, investigations on vector competence for *T. haneyi* have not been reported yet [7].

### 5.2. Camels

Ticks of the genus *Hyalomma* are most commonly associated with camels in different countries and are known vectors of *Theileria*, *Babesia*, *Anaplasma*, *Rickettsia,* and *Ehrlichia* spp. [39].

In Egypt, three tick genera were identified in infested camels (*Hyalomma*, *Rhipicephalus*, and *Amblyomma*) [40], including different species of ticks such as *Hyalomma dromedarii*, *Hyalomma rufipes*, *Hyalomma truncatum*, *Hyalomma anatolicum excavatum*, and *Hyalomma impeltatum*. In addition, *Rhipicephalus annulatus*, *Rhipicephalus sanguineus*, *Rhipicephalus pulchellus*, *Amblyomma gemma*, *Amblyomma lepidum*, and *Amblyomma variegatum* were also found [38,41].

## 6. Diagnosis of Equine and Camel Piroplasmosis

Accurate identification of the causative agents of EP and CP using serological and molecular investigative approaches is crucial for the prevention and treatment of these diseases in endemic and non-endemic areas [21]. The diagnosis of piroplasmosis only based on clinical signs is not specific and cannot differentiate between the causative agents of piroplasmosis [42]. Also, microscopical examination (ME) of blood smears has limited utility due to its low sensitivity, particularly in carrier animals with low parasitemia [43]. An additional and important limitation of these two diagnostic approaches is that they cannot identify and genetically characterize species of *Babesia* and *Theileria* spp. infecting equines and camels.

In addition, serological diagnosis (IFA and ELISA techniques) was used mainly to detect chronically infected cases; thus, they may have more epidemiological than clinical value [32,44,45]. Although generally more specific, these two methods also have limitations, including low sensitivity and specificity in the case of IFA, and the need for specific and costly equipment and reagents for ELISA. Again, none of these methods informs on the exact species or the phylogenetic relationships among species and strains of parasites involved in the infections.

Some of these drawbacks can be overcome using highly sensitive and specific DNA amplification methods, such as PCR followed by sequencing of the amplicons, which can be used at any phase of infections with piroplasm spp., including the prepatent and chronic stages [46]. In addition, ticks can also be more accurately identified and classified using PCR/sequencing approaches.

### 6.1. Equines

A few small-scale surveys using conventional PCR (cPCR) for the diagnosis of EP in Egypt have been performed so far [44,45]. Importantly, effective treatments and prevention of EP depend on the differentiation between *T. equi* and *B. caballi* [47]. Molecular approaches, such as PCR, might be useful tools for determining the infectious state of a clinical suspect, preventing infection transmission or unnecessary treatments with potentially harmful side effects [48]. However, in mixed infection cases, uniplex (u) PCR is considered time consuming and expensive when applied to numerous samples with mixed infection with *Babesia* and *Theileria* spp. [49]. PCR combined with Reverse Line Blot (RLB) hybridization is a robust technique that can overcome this problem to a large extent, since up to forty different tick-borne pathogens can be detected simultaneously [50,51]. However, this technique is considered expensive and requires well-trained operators and specialized equipment [49]. In Egypt, multiplex (m) PCR was applied to equine samples to detect the two causative agents of EP simultaneously [7].

### 6.2. Camels

Most previous studies for screening of *Piroplasma* spp. infecting camels used ME or PCR without performing sequencing and phylogenetic analysis for the parasite’s diagnosis. Usually, these studies focused only on screening one or two *Piroplasma* spp. [32]. In addition, other studies investigated multiple *Piroplasma* spp. infections in camels using molecular diagnostic methods, followed by sequencing [9,17,18,20].

## 7. Historical Overview of Equine and Camel Piroplasmosis in Egypt

### 7.1. Equine

**Figure 1:**
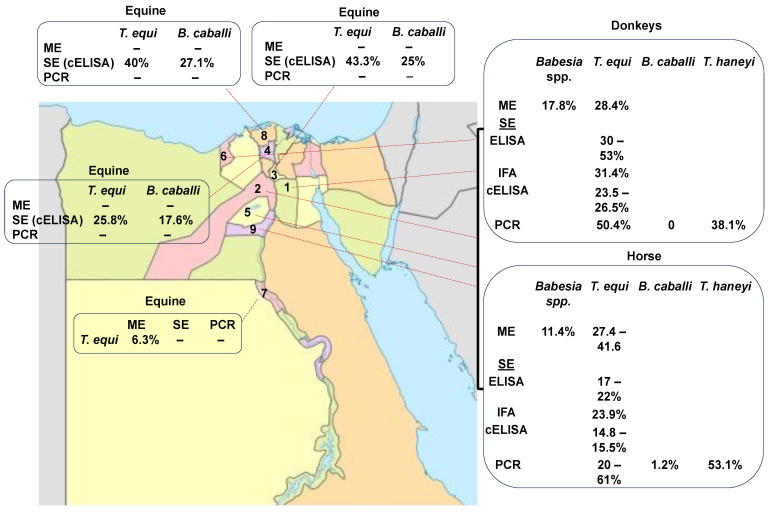
Prevalence rate of EP in different geographical regions of Egypt according to the microscopic analysis (ME), serological examination (SE), and PCR. (1. Cairo, 2. Giza, 3. Qalubia, 4. Menofia, 5. Fayom, 6. Alexandria, 7. Assiut, 8. Kafr Elsheikh, and 9. Bani Suif).

Equine piroplasmosis has been currently reported in different geographic regions of Egypt (Assiut, Cairo, Giza, Qalubia, Kafr Elshiekh, Menofia, Alexandria, Ismailia, Faiyum, Al-Beheira, Matruh, and Beni Suef) (Figure 1). In the past, the detection of the piroplasms in Egypt depended mainly on ME [52]. After that, serological studies based on IFAT revealed exposure of equines to *T. equi* [44,53] and *B. caballi* parasites [44] in the Cairo and Giza regions of Egypt. More sensitive serological methods, such as indirect (i) ELISA, also revealed the presence of *T. equi* in horses and donkeys in Egypt [44,45,54]. In addition, a competitive (c) ELISA based on the EMA-1 recombinant protein revealed the presence of *T. equi* in horses and donkeys. However, a cELISA based on RAP-1 failed to detect *B. caballi* in Egyptian equines in Cairo and Giza [44], as well as in South Africa, [55]. Possibly, given the sequence variability found among the *B. caballi* RAP-1 proteins among distinct strains from different countries, it is possible that RAP-1-based serological methods, as currently designed, are not capable of effectively detecting *B. caballi* infections worldwide.

Molecular techniques, such as PCR, have also recently been used to investigate the presence of *T. equi* in horses in the country [45,53,54]. Molecularly, *T. equi* and *B. caballi* were detected in horses and donkeys in Egypt [7,44]. In addition, *T. haneyi* was detected recently, for the first time, in horses and donkeys from Alexandria, Monufia, Ismailia, Giza, Faiyum, Beni Suef, and Cairo in Egypt [7]. Combined serology and molecular results have shown that EP, caused by *T. equi*, *B. caballi,* and *T. haneyi*, is widespread in several governorates of Egypt (Table 2 and Figure 1).

Altogether, the data collected using microscopic, serological, and molecular methods have revealed a wide prevalence of EP in Egypt (Table 2 and Figure 1) [7,32,44,45,53,54,56,57,58]. The currently available data show that the incidence of *T. equi* by microscopic analysis ranged between 11 and 38.9% in horses in Cairo and Giza. Moreover, in donkeys, EP ranged from 17.8 to 24.8% in Cairo and Giza.

Serological studies revealed that the incidence of *T. equi* ranged from 23 to 50%, 17.9 to 30%, and 14.8 to 36.5% in horses using IFA, iELISA, and cELISA, respectively. Consistently, in donkeys, the serological prevalence of *T. equi* was 31.4%, 53.4%, and 23.5–25.6% using IFA, iELISA, and cELISA, respectively.

Based on molecular techniques, the overall prevalence of *T. equi* in horses ranged from 20 to 61.9%. and 13 to 50%. in donkeys. The prevalence of *B. caballi* was 1.2–19.3% in horses and 0–15.7% in donkeys.

The recently identified *T. haneyi* was also detected in Egypt, with an incidence of 53.1% in horses and 38.1% in donkeys [7].

The wide range of variations in prevalence is shown in Table 2, which may be due to the use of different diagnostic methods with different sensitivities and specificities and/or other differences among the sets analyzed, including different sample sizes and factors associated with the diversity existing among the distinct geographic areas studied. These observations highlight the fact that standardized and systematic surveys on EP have not been performed so far in Egypt. The serological prevalence of EP caused by three distinct agents (*T. equi*, *B. caballi,* and *T. haneyi*) remains unknown. In addition, there are no commercially available or standardized enzymatic immunoassays based on crude, purified, or recombinant antigens derived from Egyptian strains of *T. equi*, *B. caballi,* or *T. haneyi* for the rapid detection of chronically infected animals affected by EP using serological approaches.

**Table 2:** The prevalence of EP in different governorates of Egypt using different diagnostic methods.

Host	Method	Year	Governorates	Sample Size	Parasite	Prevalence	Reference
Horses	ME	2003	Different localities	18	*B. equi*	38.9%	[53]
	IFA					50%	
	PCR					77.8%	
Horses	ME	2011	Not detected	100	*T. equi*	18%	[54]
Horses	ME	2013	Giza	149	*T. equi*	41.6% (Males 36.2% females 5.4%)	[58]
Horses	ELISA	2015	Cairo and Giza	50	*T. equi*	22% 30%	[56]
Donkeys				50			
Horses	ME	2016	Cairo and Giza	139	*Babesia* spp.	11.4%	[45]
Donkeys						17.8%	
Horses	IFA			88	*T. equi*	23.9%	
Donkeys				51		31.4%	
Horses	cELISA			88	*T. equi*	14.8%	
Donkeys				51		23.5%	
Horses	ME	2016	Cairo and Giza	168	*T. equi*	27.4%	[45]
Donkeys				133		24.8%	
Horses	nPCR			168	*T. equi*	61.9%	
Donkeys				133		50.4%	
Horses	cELISA			168	*T. equi*	15.5%	
Donkeys				133		25.6%	
Horse	iELISA			168	*T. equi*	17.9%	
Donkeys				133		53.4%	
Horses	ME	2018	Cairo and Giza	141	*T. equi*	5.56%	[57]
Donkeys							
				250			
Mules				5			
Horses Donkeys Mules	PCR			45	*T. equi*	30%	
				50			
				5			
Horses	cELISA	2020	Giza, Qalubia, Kafr, Elshiekh, and Menofia	370	*T. equi,*	39%,	[32]
					*B. caballi*	11%	
Donkeys				150	*T. equi,*	30.6%	
					*B. caballi,*	42%	
Horses	mPCR	2021	Alexandria, Monufia, Ismailia, Giza, Faiyum, Beni Suef, and Cairo.	79	*T. equi*	20.3%	[7]
					*B. caballi*	1.2%	
					*Mixed*	2.5%	
Donkeys				76	*T. equi*	13.1%	
					*B. caballi*	0	
					Mixed	1.%	
Horse	cPCR			79	*T. haneyi*	53.1%	
Donkeys				76	*T. haneyi*	38.1%	
Horses	cPCR	2022	AL-Faiyum, AL-Giza, Beni-Suef, Al-Menufia, Al-Beheira, and Matruh	8	*Piroplasma* spp.	0	[59]
Donkeys				22			

### 7.2. Camel

**Figure 2:**
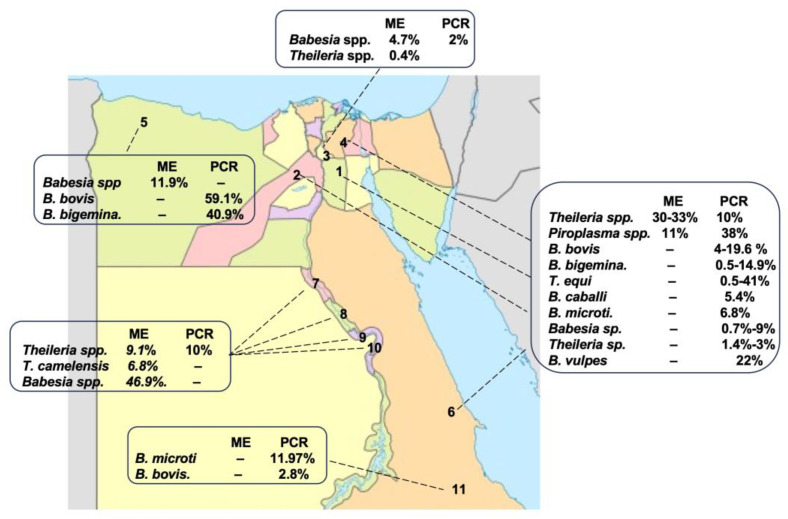
Prevalence rates of CP in different geographical regions of Egypt according to the ME, serological examination (SE), and PCR. 1. Cairo, 2. Giza, 3. Qalubia, 4. Sharkia, 5. Matruh, 6. Red sea, 7. Assiut, 8. Suhag, 9. Qena, 10. Luxur, and 11. Halayb w Shalaten.

Camel piroplasmosis has been reported in different regions of Egypt, Cairo: Giza, and Assiut—upper Egypt; Qalubia-Halayeb and Shalaten—Northern West Coastal zone; Qena and Luxor—Sharika Suhag and the Red Sea (Figure 2). First, the detection of CP was mainly dependent on ME [60,61,62,63], which reported the infection of camels with *Theileria* spp., *T. camelensis*, and *Babesia* spp. with different infection rates, such as *Theileria* spp. (9.1–33%), *T. camelensis* (6.8%), and *Babesia* spp. (46.9%). After that, a combination of ME and a molecular method (PCR) was used to obtain more accurate detection results [10,17,64,65]. Combined microscopical and molecular results have shown that CP is caused by *Theileria* spp., *T. camelensis*, *B. bovis*, *B. bigemina*, *T. annulate*, *T. ovis*, *T. equi*, *B. caballi*, *B. vulpes*, *Babesia* sp. *Theileria* sp., and *B. microti*, and it is widespread in several governorates of Egypt [9,10,17,18,20,40] (Table 3 and Figure 2). It was found that camel can be infected with different *Piroplasma* spp, suggesting infestations by different competent vectors. Overall, these data together suggest that camels should be screened for other species of *Babesia* and *Theileria* spp. that were not detected before via PCR using specific primer sets, followed by sequencing, in order to confirm the results.

## 8. Current Control Methods

The application of methods for control is important in order to improve the well-being of the animals, prevent clinical disease, and eliminate the risk of disease due to the presence of parasite reservoirs that may affect other species, including humans, via tick transmission or by other agents. Therefore, the control of piroplasmosis is crucial in order to secure food resources, improve the productivity of farm animals, and prevent the spread of these infections to humans and other animal species. The most common control protocols for the control of equine and camel piroplasmosis used in different countries are based on chemotherapy and vector control. Ideally, a similar combination of different control methods should also be applied in Egypt.

### 8.1. Treatments of Piroplasmosis Using Imidocarb Dipropionate and Diminazene Aceturate

Piroplasm-infected animals can be treated with an antiprotozoal drug, such as imidocarb (ID). The infected animals under treatment should be separated from the herd for the entire length of treatment. In the case of infection by the protozoan *B. caballi*, it is recommended that a veterinarian should give two injections of ID 24 h apart. The typical dosage range is 2 or 2.5 mg/kg of body weight. In case of infection by *T. equi*, the treatment should consist of four injections at 72 h intervals because this parasite is more resistant to treatment with this drug at a dosage of 4 mg/kg [66]. *Theileria haneyi* does not seem to be susceptible to ID; however, the co-infection of horses with *T. equi* reduces the effectiveness of ID against *T. equi*. So, it has been suggested that the global importance of *T. haneyi* to equine health may be due to its resistance to ID and its interference with *T. equi* clearance by ID in some co-infected horses [34,35].

Diminazene aceturate has been used with success against *T. equi* and *B. caballi* at a dose of 3.5 mg/kg IM every 48 h for two treatments [67].

### 8.2. Supportive Treatment

Supportive treatment is recommended, particularly in valuable animals. This approach may include the use of anti-inflammatory drugs, corticosteroids, and fluid therapy in severe cases. Blood transfusions may be lifesaving in very anemic animals, but this approach carries the risk of the transmission of other pathogens if the blood is not properly tested [66].

### 8.3. Tick Control

Tick management techniques or acaricides can help reduce tick loads, which can lessen transmission rates. Piroplasms cannot be prevented by chemical tick control alone, as they are useful only in reducing tick burdens, which can lower transmission rates [68]. Acaricides are widely considered to be the mainstay of tick control and management. Currently available acaricides include organophosphates (OP) (chlorfenvinphos, chlorpyrifos, coumaphos, and diazinon), synthetic pyrethroids (SP) (cypermethrin, deltamethrin, flumethrin, and permethrin), amidines (amitraz), and phenylpyrazole (fipronil). In addition, injectable forms of macrocyclic lactones (ML), avermectin (ivermectin, doramectin), and milbemycin (moxidectin) compounds are also used [69]. Very few records describing acaricide treatment for camels in Egypt are available. Also, as we mentioned earlier, most camel imports in Egypt go directly for processing at slaughterhouses for meat production. However, the same acaricides that are used in cattle are also utilized in camels, either in spray or injectable forms (personal communication from Dr. Mohamed Ramadan, National Research Center, Egypt). Although deltamethrin and phoxim (diethyl- O-(alpha-cyanobenzylideneamino)-thiophosphate) are used in spray forms (1 mL/1 L of water for spray and 3 mL/1 L of water as a topping up) [70], diazinon is usually used to spray the walls and grounds of the farm for tick management due to its high toxicity in farm animals. It is also worth mentioning that in the case of using phoxim for tick treatment, it is recommended to treat the animal for 5 weeks in order to eliminate all different developmental stages of the ticks. Ivermectin (1 mL/50 kg) is also used subcutaneously as an injectable form. We found a single clinical study investigating the use of ivermectin (given subcutaneously at 0.2 mg/kg) [71] in camel treatments at the St. Catherine monastery, Sinai, Egypt. That study found that ivermectin was not effective against *Hyalomma* tick infestation in camels under the study conditions [71].

Spray acaricide forms of acaricides are very rarely used to treat equines. However, phoxim is the most commonly used acaricide because it is widely regarded as safe. Regarding the injectable forms, both dectomax and mactilan, which are ivermectin formulations, should be administrated intramuscularly, rather than subcutaneously, in order to avoid irritation and abscess formation in equines.

To avoid the emergence of acaricide-resistant ticks, it is better to apply the acaricide at an accurate dose. The issue of acaricide resistance is increasing, which is worrisome. To avoid the spread of ticks and babesiosis to tick-free regions, it is helpful to employ acaricidal tick control before transporting animals from tick-infested areas. On-site tick vector eradication is seldom possible, but regionally or nationally coordinated programmes may be more successful.

The use of vaccines is considered crucial for tick control and disease prevention by inducing host-acquired immunity against ticks via active immunization with different forms of either crude, purified native, or recombinant antigens derived from ticks [72]. Thus, many studies have investigated the efficacy of antigens in developing efficacious vaccines [73]. Two commercial vaccines were previously developed for use in cattle based on the Bm86 tick midgut protein, which is considered a concealed antigen, namely TickGARD Plus [74] and Gavac Plus [75], which were developed and tested in Australia and Cuba [72], respectively. Although the production of the TickGARD Plus vaccine was discontinued in Australia in 2010 due to the need for numerous applications (3–4 boosts per year), Gavac^®^ continues to be produced [76]. There are no reports available on the use of tick vaccines in equines or camels in Egypt.

### 8.4. Vaccinations

Live vaccines based on attenuated parasites are only used in cattle against *B. bovis* and *B. bigemina* in other endemic areas worldwide [66]. Currently, there are no approved vaccines for EP and CP in Egypt and elsewhere. There is a need to develop and use vaccines to prevent the acute form of the disease and, if possible, to block transmission of the parasites by ticks. The fact that different species of parasites can cause the disease indicates the need for several types of effective live vaccines or single vaccines, including protective antigens derived from different pathogens. However, more research is needed in order to identify protective blood stages or transmission-blocking antigens and develop such vaccines.

## 9. Piroplasmosis Preventive Measures

Piroplasmosis is a blood-borne disease. The following guidelines have been suggested in order to help protect animals from developing these diseases [77]:

1. When administering injections into a vein, muscle, or skin, always use a sterilized needle and syringe.
2. Between each horse, clean all surgical, dental, and tattoo equipment. Before disinfection, be careful to remove all dirt and blood with soap and water.
3. Use only commercially authorized blood and blood products.
4. Each time a multi-dose pharmaceutical bottle is punctured, use a sterile needle.
5. Monitor ticks on the animal’s body regularly. If ticks are discovered, speak with a veterinarian about the most effective tick-prevention strategies in the region.
6. If a horse or camel exhibits symptoms of fever, jaundice, reduced appetite, or weight loss, call a veterinarian.
7. Remove detected ticks.

## 10. Concluding Remarks

The current situation of piroplasmosis in Egypt discussed in this study suggests an urgent need for the development of novel, sensitive, accurate, and accessible methods for the serological and molecular diagnosis of piroplasmosis. If possible, point-of-care diagnostic methods are also desirable. The diagnostic methods of choice should be properly validated and standardized and made available for large-scale epidemiological studies, which need to be carefully designed in order to obtain an accurate picture of the incidence and impact of piroplasmosis in Egypt. Also, field and lab personnel should be adequately trained in the application and interpretation of such studies.

Preventive control methods, such as vaccines, would be ideal, but these are currently unavailable. Developing novel vaccines will require studies on the protective mechanisms involved in the resolution of piroplasmosis in surviving animals and the definition of correlates of protection. Protective antigens (available either in recombinant, native, DNA, or RNA formats) derived from the more important agents of piroplasmosis should also be identified and tested, and then effective vaccines and vaccine delivery methods should be devised. These considerations also apply to the development of novel and effective anti-tick vaccines. While vaccines are developed, disease control should be approached using distinct methods, such as tick control, drug treatments, and animal management strategies. Taking into account that drug treatments might not be fully efficient, may lead to resistance, or be toxic for animals and humans consuming animal products, it will also be important to investigate novel, safe, and effective drugs that can control piroplasmosis.

Altogether, these will require concerted efforts by governmental agencies, stakeholders, and health providers, including veterinarians, researchers, and field workers, but awareness of the importance of these diseases in these sectors should also be fostered. Finally, and not less importantly, these efforts will require securing adequate and consistent financial support for the development of surveys and basic research toward improving the tools required for the diagnosis and treatment of this disease. This aspect can be covered by either central or local governments, stakeholder associations, national and international grants, and other resources. These suggested measures, if successful, will almost certainly result in improved animal and human health and in securing highly needed food resources in the country and the region in general.

## Figures and Tables

**Table 1:** Keywords used for searching different databases.

Pathogens and Diseases	Animals	Methods	Country	Databases
Tick-borne diseases *Babesia* Babesiosis *Theileria* Theileriosis	Camel Equines Horses Donkeys	Microscopical Serological Molecular PCR	Egypt	PubMed Scopus ScienceDirect Egyptian Knowledge Bank Google Scholar

**Table 3:** The prevalence of CP in different regions of Egypt determined using microscopical and molecular techniques.

Method	Year	Governorates	Sample Size	Parasite	Prevalence	Reference
ME	1992	Cairo and Giza	200	*Theileria* spp.	30%	[63]
ME	1998	Cairo	74	*Theileria* spp.	33.3%	[62]
ME	2011	Upper Egypt	224	*T. camelensis*	6.8%	[61]
ME	2014	Assiut Upper Egypt	89	*Babesia* spp.	46.9%	[60]
				*Theileria* spp.	9.1%	
ME	2015	Giza	243	*Theileria* spp.	30.9%	[64]
PCR					10%	
ME	2016	Northern West Coastal zone	331	*Babesia* spp.	11.9%	[10]
PCR				*B.bovis*	59.1%	
				*B. bigemina*	40.9%	
ME	2018	Qalubia	700	*Babesia* spp. *Theileria* spp.	4.7% 0.4%	[65]
PCR			100 (negative ME)	*Babesia* spp	2%	
nPCR	2021	Halayeb and Shalaten	142	*B. bovis*	2.81%	[18]
ME	2023	Cairo, Giza, Qalubya, Sharika Suhag, and Red Sea	531	*Piroplasma* spp.	11%	[16,17]
cPCR				*Babesia/Theileria* spp.	38%	
mPCR				*T. equi* (SI)	41%	
				*T. equi* (Mixed)	0.5%	
				*B. caballi* (Mixed)	5.4%	
				*B. bovis* (SI)	4%	
				*B. bovis* (Mixed)	5%	
				*B. bigemmina* (Mixed)	0.5%	
nPCR				*B. vulpes*	22%	
				*Babesia* sp.	9%	
				*Theileria* sp.	3%	
nPCR	2021	Halayb and Shalaten	142	*B. microti*	11.97%	[9]
PCR	2022	Giza, Asyut, Sohag, Qena, Luxor, and the Red Sea	148	*B. bovis*	19.6%	[20]
				*B. bigemina*	14.9%	
				*Babesia* sp.	0.7%	
				*Theileria* sp.	1.4%	
				*T. equi*	0.7%	
nPCR	2023	Cairo and Giza	133	*B. microti*	6.8%	[40]

SI: single infection.

## Data Availability

Not applicable.
